# Hetero and homo α,ω‐chain‐end functionalized polyphosphazenes

**DOI:** 10.1002/pol.20220066

**Published:** 2022-04-08

**Authors:** Paul Strasser, Oliver Plavcan, Edip Ajvazi, Helena Henke, Oliver Brüggemann, Ian Teasdale

**Affiliations:** ^1^ Institute of Polymer Chemistry Johannes Kepler University Linz Linz Austria; ^2^ Centre for Additive Manufacturing University of Nottingham, Jubilee Campus, Wollaton Road Nottingham, NG8 1BB UK

**Keywords:** chain‐end functionalization, inorganic polymers, polyphosphazenes, soft materials, telechelic polymers, thiol‐ene addition

## Abstract

The control of chain‐ends is fundamental in modern macromolecular chemistry for directed one‐to‐one bioconjugation and the synthesis of advanced architectures such as block copolymers or bottlebrush polymers and the preparation of advanced soft materials. Polyphosphazenes are of growing importance as elastomers, biodegradable materials and in biomedical drug delivery due to their synthetic versatility. While controlled polymerization methods have been known for some time, controlling both chain‐ends with high fidelity has proven difficult. We demonstrate a robust synthetic route to hetero and homo α,ω‐chain‐end functionalized polyphosphazenes via end‐capping with easily accessible, functionalized triphenylphosphine‐based phosphoranimines. A versatile thiol‐ene “click”‐reaction approach then allows for subsequent conversion of the end‐capped polymers with various functional groups. Finally, we demonstrate the utility of this system to prepare gels based on homo α,ω‐chain‐end functionalized polyphosphazenes. This development will enhance their progress in various applications, particularly in soft materials and as degradable polymers.

## Graphical abstract

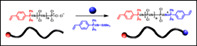

## 1 INTRODUCTION

Polyphosphazenes are a class of inorganic–organic hybrid polymers^1^ offering a diverse set of properties and applications in various fields, including self‐assembly,^2^, ^3^ high‐performance elastomers^4^, ^5^ to biomedical applications such as stent coatings,^6^ injectable hydrogels,^7^ vaccine adjuvants,^8^, ^9^, ^10^ nanomedicine,^11^ or polymer therapeutics.^12^, ^13^ This considerable variability is mainly rooted in their unique synthesis via the macromolecular precursor poly(dichloro)phosphazene ([NPCl_2_]_*n*_). The facile post‐polymerization substitution reaction of [NPCl_2_]_*n*_ with suitable organic side groups yields a vast array of poly(organo)phosphazenes ([NPR_2_]_*n*_).^1^, ^14^ Based on the selection of organic side groups, as a homo or a mixed substitution, the properties of the polymer can be tailored to fit targeted specifications.^4^ Nevertheless, polymer properties are also governed by their macromolecular structure and architecture, with modern polymer chemistry utilizing controlled polymerization approaches with high end‐group fidelity.^15^, ^16^ Furthermore, functional chain‐ends on polymers open the door to their use in macromolecular engineering, surface functionalization, bioconjugation, and advanced materials.

The macromolecular [NPCl_2_]_*n*_ intermediate is traditionally prepared via a thermal ring‐opening polymerization. However, this allows only for polymers of poorly controlled molecular weight and broad dispersity.^17^ With the development of ambient temperature polymerization routes, such as the PCl_5_ initiated living cationic polymerization of trichlorophosphoranimine (Cl_3_PNSiMe_3_), increased control over the polymerization process could be achieved, enabling the regulation of the molecular weight and dispersity.^18^, ^19^, ^20^ Furthermore, one‐pot synthesis pathways have been developed with in situ polymerization of the monomer occurring directly after its formation.^21^, ^22^ Alternatively, dichlorophosphoranes have also been shown to initiate the polymerization of Cl_3_PNSiMe_3_, leading to a monodirectional growth of the polymer chain and a defined α‐chain‐end.^23^, ^24^ Building on these findings, our group reported the synthesis of well‐defined α‐chain‐end functionalized polyphosphazenes based on chlorinated, commercially available triphenylphosphines with functional groups in a simple one‐pot approach.^25^ Different routes to enable functional and terminal groups at the chain‐ends of poly(alkyl/aryl)phosphazenes are reported.^26^ However, ths limited in terms of the scope of materials that can be produced. Current routes to telechelic versions of the versatile [NPCl_2_]_*n*_ precursor require extensive, time‐consuming synthesis of alkoxy/aryloxy organophosphoranimines and have thus not found widespread use.^27^, ^28^ Furthermore, while these routes can be used to terminate [NPCl_2_]_*n*_ polymers, initiation with such species gives inconsistent molecular weight control and broad dispersities, thus hindering the preparation of bifunctional chain‐end functionalized [NPCl_2_]_*n*_.^28^, ^29^

Herein we develop a robust, easily accessible and broadly applicable synthesis of telechelic and heterotelechelic polyphosphazenes, extending the α‐chain‐end functionalized [NPCl_2_]_*n*_ to give hetero and homo α,ω‐chain‐end functionalized polyphosphazenes. Allcock et al. previously prepared polystyrene‐block‐[NPCl_2_]_*n*_ via termination of a living [NPCl_2_]_*n*_ polymer with phosphine capped polystyrene.^30^, ^31^ After converting the terminal phosphine to a phosphoranimine, it was possible to cap the living chain‐end of [NPCl_2_]_*n*_ with the polystyrene. Herein we adapt and combine these separately developed procedures, designing a novel, functionalized phosphoranimine chain‐end‐capper to give α,ω‐chain‐end functionalized polyphosphazenes. Such simple access to well‐defined α,ω‐chain‐end functionalized polymers will encourage the development of higher molecular architectures of polyphosphazenes and aid their advancement in advanced materials.

## 2 EXPERIMENTAL SECTION

### 2.1 Materials and instrumentation

Chemicals were purchased from different vendors and used as received unless stated otherwise. Hexachloroethane, 2,2‐dimethoxy‐2‐phenylacetonephenone (DMPA), 4‐(diphenylphosphino)styrene, 6‐mercapto‐1‐hexanol, lithium bis(trimethylsilyl)amide, phosphorus trichloride, thionyl chloride, trimethylsilyl azide, trimethylpropane tris(3‐mercaptopropionate), 4‐diphenylphosphanylbenzoic acid 2‐(trimethylsilyl)ethyl ester solution (0.5 M in THF) and 2‐(boc‐amino)ethanethiol were purchased from Sigma Aldrich. Anhydrous dichloromethane and anhydrous tetrahydrofuran, as well as Celite® (325 Mesh powder) were bought from Alfa Aesar, *N*‐(3‐aminopropyl)morpholine from TCI, triethylamine from Merck, and 2,2′‐azobis(2‐methylpropionitrile) from Arcos organics. Anhydrous ethanol and dichloromethane were purchased from ChemLabs, trifluoroacetic acid from Honeywell Fluka, tetrahydrofuran, diethyl ether and ethanol from VWR and chloroform‐d from Eurisotop.

Et_3_N was distilled and dried over molecular sieves (3 Å) prior to use, and air and moisture sensitive syntheses were carried out in an argon filled glovebox (MBRAUN) under inert atmosphere. Dialysis was carried out with Spectrum™ Spectra/Por™ RC membrane tubing with different molecular weight cut‐offs. EtOH used as a solvent was recycled by distillation. Thiol‐ene reactions were performed at 5 °C in a photochemical reactor from Rayonet using UV‐light with a wavelength of 365 nm. NMR spectra were recorded on a Bruker Avance III 300 at 25 °C in CDCl_3_. Size exclusion chromatography (SEC) was performed on a Viscothek GPCmax equipped with a PFG column from PSS (Mainz, Germany) (300 mm × 8 mm, 5 μm particle size) and DMF containing 10 mM LiBr as the mobile phase at a flow rate of 0.75 ml min^−1^ at 60 °C. The samples were filtered through 0.2 μm PTFE syringe filters prior to injection and their molecular weights were determined via a multiple detector calibration of the light scattering, refractive index, and viscosity detectors calibrated with a polystyrene standard from PSS.

### 2.2 Synthesis of *N*‐(trimethylsilyl)‐trichlorophosphoranimine (Cl_3_PNSiMe_3_)

The monomer for polyphosphazene polymerization, Cl_3_PNSiMe_3_, was synthesized according to slightly adapted literature procedure.^35^ Briefly, lithium bis(trimethylsilyl)amide (25.08 g, 0.15 mol, 1 eq.) was dissolved in 500 ml anhydrous diethyl ether under argon and cooled to 0 °C. Phosphorus trichloride (13.07 ml, 0.15 mol, 1 eq.) was added dropwise over the course of 1 h and stirred for another 30 min at 0 °C. Subsequently, thionyl chloride (12.08 ml, 0.15 mol, 1 eq.) was added again over the course of 1 h and stirred for additional 30 min at 0 °C. The reaction mixture was filtered through dry Celite® and the solvent was carefully removed under reduced pressure at room temperature. The final product was obtained via vacuum distillation at 40 °C as a clear and colorless liquid (22.01 g, yield 65%) and stored under argon at −35 °C. The corresponding NMR spectra are depicted in Figures S10 and S11.

^1^H‐NMR (300 MHz, CDCl_3_, δ): 0.17 ppm (s, 9H); ^31^P{^1^H}‐NMR (121 MHz, CDCl_3_, δ): −54.66 ppm.

### 2.3 Synthesis of end‐capper, **1**

Trimethylsilyl azide (1.39 g, 12.07 mmol, 4 eq.) in 5 ml anhydrous CH_2_Cl_2_ was added to a solution of 4‐(diphenylphosphino)styrene (0.87 g, 3.08 mmol, 1 eq.) in 20 ml anhydrous CH_2_Cl_2_ and stirred at room temperature in the glove box for 4 days. Afterwards, CH_2_Cl_2_ and residual azide were evaporated under reduced pressure resulting in phosphoranimine **1** as a yellow, waxy solid (0.76 g, 2.68 mmol, 87%). The product was redissolved in diglyme or CH_2_Cl_2_ for long term or short term storage, respectively. The corresponding NMR spectra are depicted in Figures S1 and S2.

^1^H‐NMR (300 MHz, CDCl_3_, δ): −0.18 (s, 1.5H), −0.05 (s, 8H), 5.35 (d, ^3^J_HH_ = 11 Hz, 1H), 5.84 (d, ^3^J_HH_ = 17.6 Hz, 1H), 6.74 (dd, ^3^J_HH_ = 10.9 Hz and ^3^J_HH_ = 17.5 Hz 1H), 7.36–7.71 ppm (m, 14H); The peak at around 0.27 ppm is assigned to residual N_3_‐SiMe_3_ and the peak at 0.17 ppm is assigned to the hydrolyzed by‐product HO‐SiMe_3_. ^31^P{^1^H}‐NMR (121 MHz, CDCl_3_, δ): 0.78 ppm.

### 2.4 Synthesis of homo‐α,ω‐chain end functionalized poly(*N*‐(3‐aminopropyl)morpholine)phosphazene, P3

The poly(organo)phosphazene was synthesized adapted from typical literature procedures^25^, ^36^ at room temperature in the glove box. 4‐(Diphenylphosphino)styrene (0.58 g, 2.01 mmol, 1 eq.) and hexachloroethane (0.52 g, 2.21 mmol, 1.1 eq.) were dissolved in ≈ 1 ml CH_2_Cl_2_, mixed and stirred for 24 h. The resulting initiator (**2**) was checked via ^31^P‐NMR spectroscopy and the polymerization was performed in situ with addition of Cl_3_PNSiMe_3_ (3.16 g, 14.08 mmol, 7 eq.), dissolved ≈ 2 ml CH_2_Cl_2_. After stirring for 12 h the synthesized α‐[NPCl_2_]_*n*_ (**P1**) was checked again via ^31^P‐NMR spectroscopy and subsequently end‐capped by dissolving the end‐capper (1.13 g, 3.02 mmol, 1.5 eq.) in 8 ml anhydrous CH_2_Cl_2_ and adding the solution to the reaction mixture. After stirring for 24 h, the product, α,ω‐[NPCl_2_]_*n*_ (**P2**), was verified by ^31^P‐NMR spectroscopy and the macrosubstitution reaction was performed. *N*‐(3‐aminopropyl)morpholine (6.17 ml, 42.24 mmol, 21 eq.) and Et_3_N (11.78 ml, 84.49 mmol, 42 eq) were dissolved in around 40 ml anhydrous THF and the α‐ω‐[NPCl_2_]_*n*_ solution in CH_2_Cl_2_ was added dropwise resulting in a turbid, white solution after some minutes. The reaction was stirred for 24 h, removed from the glove box, filtered and the solvent was removed. The crude product was purified by dialysis against ethanol for 48 h (1 kDa MWCO) and dried under vacuum at 45 °C for 24 h yielding a waxy, orange‐yellow product (4.55 g, 1.343 mmol, 67%). The corresponding NMR spectra are depicted in Figures S4, S5, and S12–S15.

Dichlorodiphenyl(4‐vinylphenyl)‐λ^5^‐phosphorane (**2**): ^31^P{^1^H}‐NMR (121 MHz, CDCl_3_, δ): 63.33 ppm.

α‐Poly(dichloro)phosphazene (**P1**): ^31^P{^1^H} NMR (121 MHz, CDCl_3_, δ): −18.19, 8.0, 19.50 ppm.

α‐ω‐Poly(dichloro)phosphazene (**P2**): ^31^P{^1^H} NMR (121 MHz, CDCl_3_, δ): −18.21, 18.01, 19.48 ppm.

Poly(*N*‐(3‐aminopropyl)morpholine)phosphazene (**P3**): ^1^H NMR (300 MHz, CDCl_3_, δ): 1.67 (br, 26H), 2.41 (br, 80H), 2.88 (br, 26H), 3.68 (br, 55H), 5.43 (br, 2H), 5.88 (br, 2H), 6.74 (br, 2H), 7.59 (br, 28H) ppm; ^31^P{^1^H} NMR (121 MHz, CDCl_3_, δ): 3.57, 6.12, 8.22, 8.90, 11.25 ppm.

### 2.5 Diol functionalization of polymer P3 via thiol‐ene “click”‐chemistry, P4

The synthesis was performed directly with polymer **P3**, synthesized as described above, and calculated based on the initiator amount. The polymer (705 mg **P3** polymerized from 60 mg initiator, 0.21 mmol, 1 eq.) and 2,2‐dimethoxy‐2‐phenylacetophenone (40.5 mg, 0.19 mmol, 10 w%) were dissolved in 5 ml anhydrous ethanol and 6‐mercapto‐1‐hexanol (57 μl, 0.83 mmol, 2 eq.) was added. The flask was sealed, flushed with argon for 10 min and irradiated with UV‐light under stirring at 4 °C overnight. The solvent was evaporated and the crude product dialyzed against EtOH (1 kDa MWCO) for 24 h. Finally, the solvent was evaporated again, yielding an orange‐yellow solid (352 mg, 0.1 mmol, 46%). The corresponding NMR spectra are depicted in Figures S16 and S17.

^1^H‐NMR (300 MHz, CDCl_3_, δ): 1.13–1.79 (m, 26H), 2.41 (br, 50H), 2.88 (br, 16H), 3.68 (br, 35H), 7.29–7.78 (m, 14H) ppm; ^31^P{^1^H}‐NMR (121 MHz, CDCl_3_, δ): 3.65, 6.36, 8.25, 8.91, 11.32 ppm.

### 2.6 Synthesis of hetero α,ω‐chain end functionalized poly(*N*‐(3‐aminopropyl)morpholine)phosphazene (**P6**) and its amine functionalization, **P7**

The hetero‐α,ω‐chain end functionalized poly(*N*‐(3‐aminopropyl)morpholine)phosphazene was synthesized according to the adapted synthesis of the homo‐α,ω‐chain end functionalized poly(*N*‐(3‐aminopropyl)morpholine)phosphazene described above and from literature.^25^

To synthesize the initiator **3**, 4‐diphenylphosphanly benzoic acid‐2‐(trimethylsilyl) ethyl ester (540.5 μl of 0.5 M in THF, 500 mg, 0.270 mmol, 1 eq.), and hexachloroethane (70.4 mg, 0.3 mmol, 1.1 eq.) were dissolved in ≈1 ml CH_2_Cl_2_, mixed and stirred for 3 days in the glove box. The polymerization was subsequently performed in situ by addition of monomer (3.0341 g, 13.52 mmol, 50 eq.), the reaction was stirred for 1 day and quenched by addition of the end‐capper (**1**) (203.0 mg, 0.54 mmol, 2 eq.) and additional stirring for 24 h. Finally, the macrosubstituent, *N*‐(3‐aminopropyl)morpholine, (4.8723 g, 33.78 mmol, 125 eq.) and Et_3_N (4.71 ml, 33.78 mmol, 125 eq.) were added to the reaction solution and stirred for another 24 h, the solution was filtered through filter paper, the solvent was subsequently evaporated and the product dialyzed against EtOH for 24 h (1 kDa MWCO) resulting in a light yellow solid after evaporation. In a next step the ω‐chain end of the α‐ω‐[NPR_2_]_*n*_ (**P6**) was functionalized via a thiol‐ene reaction. The polymer, 2,2‐dimethoxy‐2‐phenylacetophenone (460.8 mg, 1.8 mmol, 10 w%) and 2‐(boc‐amino)‐ethanethiol (92 μl, 0.54 mmol, 2 eq.) were dissolved in 10 ml CH_2_Cl_2_, degassed with argon for 10 min and irradiated under UV‐light under stirring at 4 °C overnight. The solvent was evaporated, the product dialyzed against EtOH overnight, the EtOH was evaporated and the product redissolved in 30 ml CH_2_Cl_2_. The solution was stirred vigorously and 6 ml TFA were added slowly, turning the solution dark brown. After 10 min, the solvent was evaporated and the product washed with MeOH by addition of 50 ml and evaporation of the solvent, this was repeated five times in total. Finally, the polymer was dialyzed against NaHCO_3_ (until neutral and no visible CO_2_ evolution, ~48 h) in MilliQ water (12 h) and EtOH (12 h) resulting in an amber colored solid after evaporation (2.2028 g, 0.13 mmol, 47%). The corresponding NMR spectra are depicted in Figures S6–S8, S18, and S19.

(**P6**): ^1^H‐NMR (300 MHz, CDCl_3_, δ): 0.10 (s, 9H), 1.66 (br, 144H), 2.41 (br, 497H), 2.88 (br, 154H), 3.68 (br, 344H), 5.30–5.60 (m, 1H), 5.78–6.05 (m, 1H), 6.65–6.85 (m, 1H), 7.32–7.97 (m, 25H) ppm; ^31^P{^1^H}‐NMR (121 MHz, CDCl_3_, δ): 3.57, 11.21 ppm.

(**P7**): ^1^H‐NMR (300 MHz, CDCl_3_, δ); ^31^P{^1^H}‐NMR (121 MHz, CDCl_3_, δ): 0.09 (s, 9H), 1.66 (br, 151H), 2.41 (br, 528H), 2.88 (br, 162H), 3.68 (br, 361H), 7.36–7.92 (m, 29H) ppm; ^31^P{^1^H}‐NMR (121 MHz, CDCl_3_, δ): 3.97, 4.85, 11.31 ppm.

### 2.7 Curing of homo bifunctional **P3** via radical polymerization

Homo α,ω‐chain end functionalized polymer **P3** (80 mg, 0.02 mmol) and 2,2′‐azobis(2‐methylpropionitrile) (4 mg, 24.36 mmol, 5 w%) were dissolved in 0.2 ml anhydrous THF in a vial, flushed with argon, sealed and placed in a water bath at 65 °C. The reaction was allowed to proceed overnight resulting in a pale‐greenish, crosslinked gel.

### 2.8 Curing of P3 via thiol‐ene crosslinking

Homo α,ω‐chain end functionalized polymer **P3** (100 mg, 0.03 mmol, 1 eq.) and trimethylolpropane tris(3‐mercaptopropionate) (18.56 mg, 0.05 mmol, 1.5 eq.) were dissolved in 0.2 ml anhydrous THF in a vial and 2,2‐dimethoxy‐2‐phenylacetophenone (5 mg, 19.51 mmol, 5 wt%) was added. The vial was flushed with argon, sealed and irradiated with UV‐light under stirring at 4 °C overnight resulting in a pale‐yellowish, crosslinked gel.

## 3 RESULTS AND DISCUSSION

**SCHEME 1:**
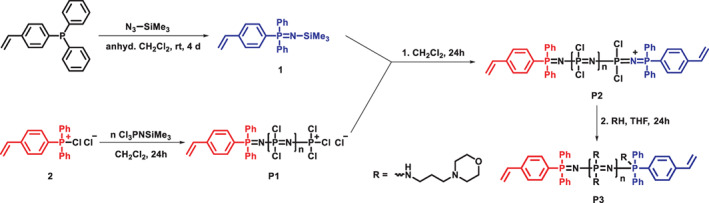
Reaction scheme for the synthesis of homo α,ω‐chain‐end functionalized α‐ω‐[NPR_2_]_*n*_ **P3**

**FIGURE 1:**
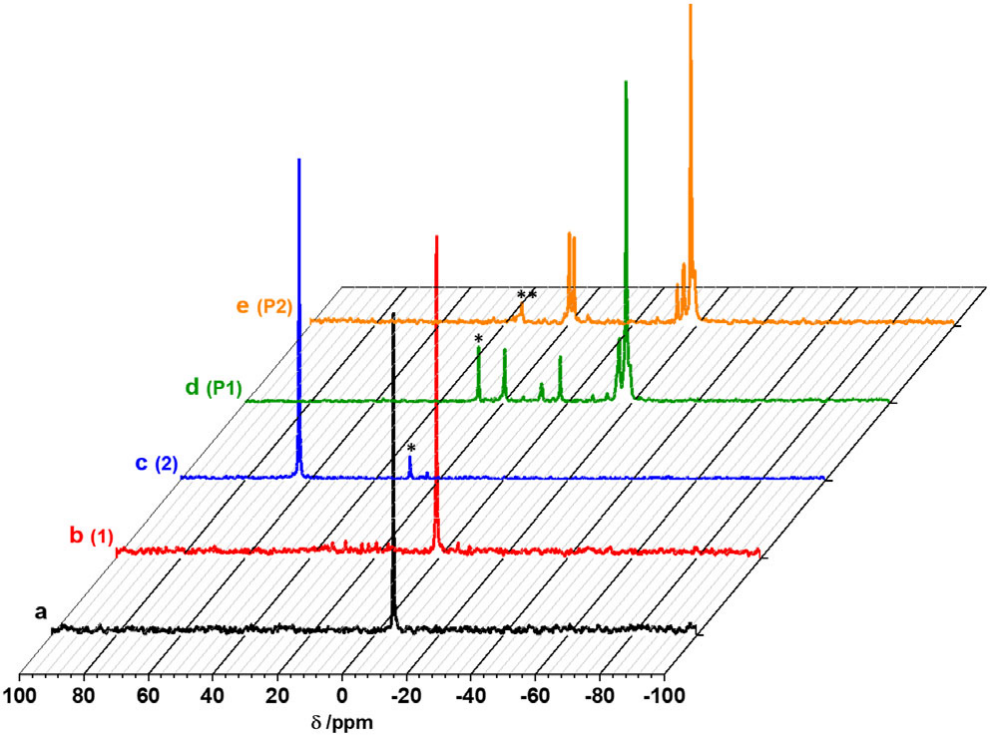
^31^P‐NMR spectra in CDCl_3_ of the different reaction steps starting from the initiator 4‐(diphenylphosphino)styrene and the PPz‐monomer toward α‐ω‐[NPR_2_]_*n*_. (a) 4‐(diphenylphosphino)styrene, (b) end‐capper **1**, (c) active initiator **2**, (d) α‐chain‐end functionalized [NPCl_2_]_*n*_ (**P1**), (e) α,ω‐chain end functionalized [NPCl_2_]_*n*_ (**P2**). * the signal around 27 ppm indicates the formation of phosphine oxide in the NMR tube during sample preparation and the measurement. ** the signal around 34.85 ppm indicates some hydrolysis of the end‐capper

A novel *N*‐organophosphoranimine end‐capper was designed and synthesized for the controlled synthesis of α,ω‐chain‐end functionalized poly(organo)phosphazene. For this purpose, a Staudinger reaction of 4‐(diphenylphosphino)styrene and trimethylsilyl azide was performed in CH_2_Cl_2_ with a fourfold excess of the azide, enabling a reasonable reaction time of 4 days at room temperature (Scheme 1). After evaporation of the solvent and the excess azide under vacuum, the resulting end‐capper **1** was isolated and verified by nuclear magnetic resonance (NMR) spectroscopy (Figure 1 and Figures S1 and S2 **)**. A shift from −5.93 ppm in the ^31^P‐NMR spectrum, for the 4‐(diphenylphosphino)styrene to 0.8 ppm for **1** can be seen (Figure 1A,B). The ^1^H‐NMR spectrum, Figure S1, shows the aromatic peaks of the phosphine along with the double bond peaks of the styrene, around 7.5 and 5.35, 5.84 and 6.74 ppm, respectively. The trimethylsilyl‐group can be seen at −0.05 ppm, however, the integral suggests a conversion of ≈85%. A small peak at 0.29 ppm further indicates some residual trimethylsilyl azide, which, however, does not interfere with the subsequent synthesis. Since the *N*‐organophosphoranimine became insoluble after prolonged periods storage as a solid, it was immediately returned to solution and stored in diglyme, a method reported and Andrianov to store [NPCl_2_]_*n*_.^17^ The stability in diglyme was tested over the course of 4 months and remained soluble and showed no changes in the ^31^P‐NMR spectroscopy (Figure S3).

The synthesis of the α‐[NPR_2_]_*n*_ was based on the previously described phosphine mediated polymerization.^23^, ^25^ To this end 4‐(diphenylphosphino)styrene was chlorinated with a small excess of hexachloroethane, yielding **2** as the initiating species for polyphosphazene polymerization. Addition of the monomer Cl_3_PNSiMe_3_, in the respective ratio to the initiator (1:7), resulted in an α‐chain end functionalized [NPCl_2_]_*n*_ (**P1**) with a chain length of *n* ≈ 7. The relatively short chain length of the polymer was selected to enable simple verification of the end‐capping by NMR‐spectroscopy (vide infra). The polymer was end‐capped at this stage with a moderate excess of the synthesized end‐capper **1**, yielding the α,ω‐chain‐end functionalized macromolecular intermediate (**P2**). The macromolecular substitution reaction with *N*‐(3‐aminopropyl)morpholine then gave the final α‐ω‐[NPR_2_]_*n*_ **P3** (Scheme 1). NB: The presence of the P(V) species in **P3** is suggested based on literature reports^27^, ^32^ but could not be detected in the ^31^P NMR spectra. The side group was chosen based on its hydrophilic character and the anticipated biodegradability of the resulting polymer, with a view to their potential future use as hydrogels.

In general, the overall synthesis was followed systematically via ^31^P‐NMR spectroscopy,^25^ starting with the chlorination of the initiator, with a shift from −5.93 to 63.33 ppm (Figure 1C). Addition of the Cl_3_PNSiMe_3_ monomer (−54.66 ppm) results in the α‐chain‐end functionalized [NPCl_2_]_*n*_ (**P1**) with the typical shift at −18 ppm and the signals for the chain ends at 19.50 and 8.09 ppm (Figure 1D). The smaller signals at 2.07 or 16.07 ppm correspond to phosphorous atoms in the proximity of the chain ends. Upon addition of **1**, the signals at 8.09 and 2.27 ppm disappear, and an additional signal at 18.01 ppm emerges (Figure 1E), indicating the linkage of the ω‐chain end to the [NPCl_2_]_*n*_ chain (**P2**). After the macromolecular substitution reaction, a broader signal for the poly(organo)phosphazene can be seen at 3.57 ppm with several smaller peaks, for example, at 8.90, 8.22, or 11.25 ppm corresponding to the chain ends, as well as the neighboring phosphorus atoms (Figure S5). In the ^1^H‐NMR of polymer **P3**, Figure S4, the signals for the morpholine side groups can be seen at 1.67, 2.41, 2.88, and 5.43 ppm, with the respective ratio of 2:6:2:4, alongside the signals of the end groups with the aromatic hydrogens at around 7.60 ppm and the double bond protons at 5.41, 5.88, and 6.74 ppm. Based on the integral ratios and the targeted chain length of *n* ≈ 7 in combination with the ^31^P‐NMR spectra, the reaction of the end‐capper onto the polymer chain and hence the formation of the novel homo α,ω‐chain end‐functionalized poly(organo)phosphazene was confirmed.

**SCHEME 2:**
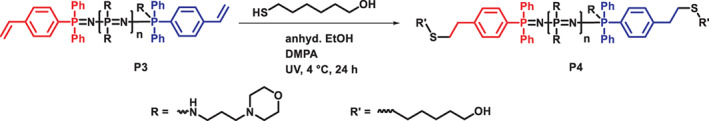
Reaction scheme for the modification of homo α,ω‐chain‐end functionalized poly(organo)phosphazene **P3** toward diol **P4** via thiol‐ene “click” reaction

**FIGURE 2:**
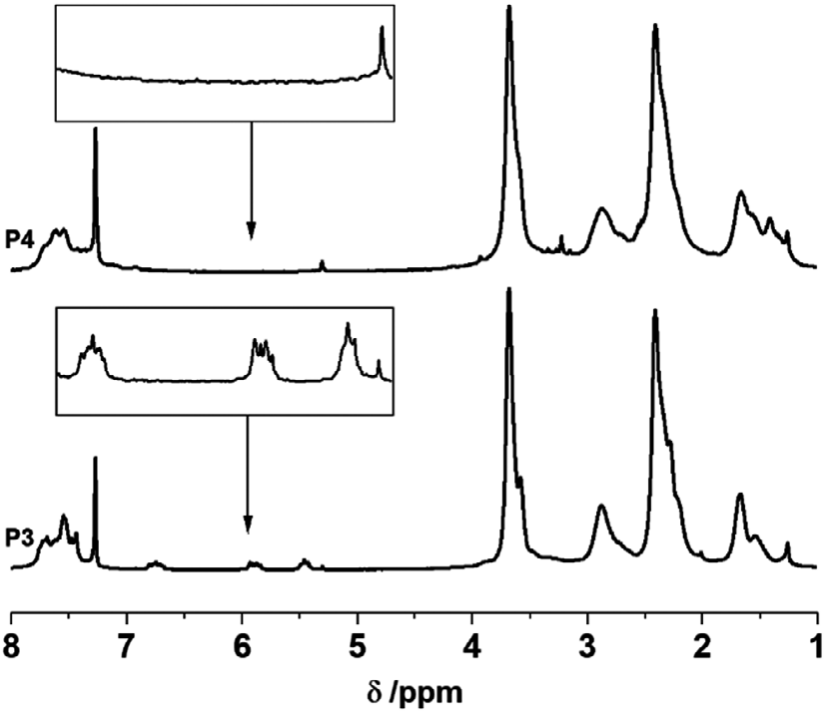
^1^H‐NMR spectra in CDCl_3_ of the α‐ω‐[NPR_2_]_*n*_ **P3** and diol‐functionalized α‐ω‐[NPR_2_]_*n*_ **P4**. The zoomed‐in regions in the boxes clearly show a disappearance of the styrene‐double bond peaks, indicating complete end‐group conversion

The double bonds of the styrene end‐groups can be used to introduce different moieties on the chain ends via thiol‐ene “click”‐reaction. As shown in Scheme 2, 6‐mercapto‐1‐hexanol can be added onto the polymer chain ends of **P3**, yielding a diol (**P4**) for further reactions. From the ^1^H‐NMR spectrum in Figure 2, the signals corresponding to the styrene double bond clearly disappear. The signals for the hexamethylene moiety can unfortunately not be unambiguously assigned due to overlapping with the protons of the morpholine side‐groups. However, a broadening of said signals can be observed. This versatile and facile reaction could be used for the insertion of a wide variety of different thiols enabling the modification of the α‐ω‐[NPR_2_]_*n*_ chain ends with different functionalities.^33^, ^34^

**SCHEME 3:**
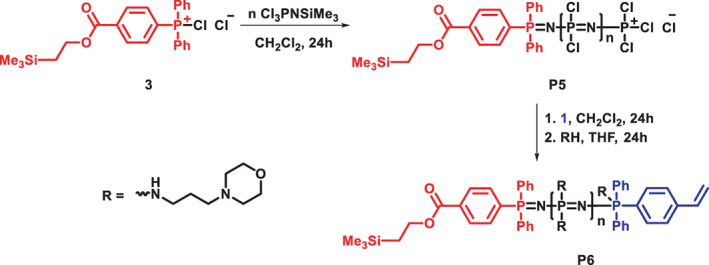
Reaction scheme for the synthesis of hetero α,ω‐chain‐end functionalized poly(organo)phosphazene **P6**

Furthermore, a hetero chain‐end functionalized polymer was synthesized in a similar fashion, as shown in Scheme 3. For this, **3** was used as the initiator, hence forming the α‐chain end [NPCl_2_]_*n*_. To show the applicability of the method for longer polymer chains, an α‐[NPCl_2_]_*n*_ with approximately 50 repeating units was synthesized by the addition of the monomer to the chlorinated initiator in a ratio of 1:50. After end‐capping the α‐[NPCl_2_]_*n*_ with the *N*‐organophosphoranimine **1**, the macrosubstitution reaction was again performed with *N*‐(3‐aminopropyl)morpholine, yielding polymer **P6**. From the ^1^H‐NMR spectrum, Figure S6, one can see the characteristic peak for the trimethylsilyl group of **3** at around 0.1 ppm and the signals arising from the aromatic carbons of both end‐groups at around 7.4–7.8 ppm. The protons from the double bonds of the styrene group can be seen at 5.41, 5.88, and 6.74 ppm, Figure S7. While the end group signals are, as expected, less well visible for the longer polymer, the ratio of the integrals fit well with 9 H for the trimethylsilyl group, approximately 1 H for each double bond signal and around 25 H for the aromatic protons. The slight deviation may be due to the lower signal‐to‐noise ratio or incomplete conversion of the ω‐chain end, nevertheless, only to a small extent. The ^31^P‐NMR spectrum, Figure S8, shows a broad peak at around 3.5 ppm characteristic for the poly(organo)phosphazene with a second peak at around 11 ppm, similar to the homo α‐ω‐[NPR_2_]_*n*_ (**P3**). Polymer **P6** was further characterized by size exclusion chromatography in DMF, as can be seen in Figure S9, showing one peak for the synthesized polymer and a molecular weight of *M*_*n*_ = 26.1 kDa with a dispersity of 1.2 according to multidetector calibration.

**SCHEME 4:**
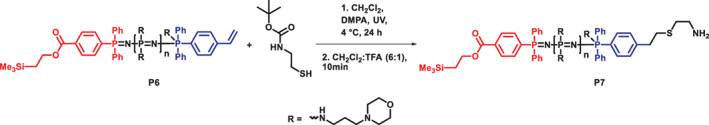
Reaction scheme for the modification of hetero α,ω‐chainend functionalized poly(organo)phosphazene **P6** toward macromolecular amine **P7** via thiol‐ene “click” reaction

Analogous to the homo chain‐end‐functionalized polymer, the styryl moieties of the hetero chain end functionalized polyphosphazenes (**P6**) were also converted via thiol‐ene “click”‐reaction, Scheme 4. To this end, 2‐(boc‐amino)ethanethiol was added and the boc‐group subsequently deprotected with TFA, resulting in polymer **P7**. The disappearance of the signals from the vinyl protons can be observed. Additionally, the presence of a free amine after deprotection was verified by a Ninhydrin‐test.

**FIGURE 3:**
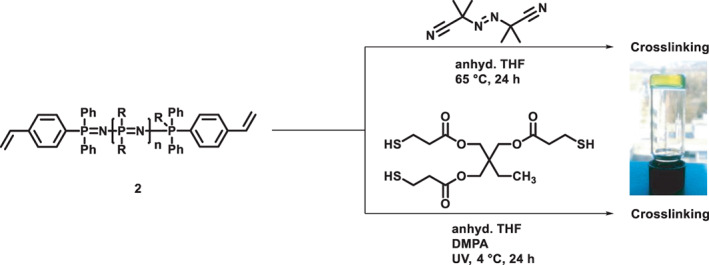
Reaction scheme for the synthesis of organogels based on the homo α‐ω‐[NPR_2_]_*n*_ via the two different approaches of direct radical polymerization with AIBN (top) and thiol‐ene reaction with a trithiol (bottom) along with an image of the formed gel

Finally, the double bonds of the synthesized macromolecular diene, polymer **P3**, were used to crosslink the homo α,ω‐chainend functionalized poly(organo)phosphazene by radical polymerization techniques either via a thiol‐ene “click”‐reaction with a crosslinker or directly by radical polymerization of the styrene end‐groups, Figure 3. For the thiol‐ene cross‐linking, the trithiol trimethylpropane tris(3‐mercaptopropionate) was dissolved along with the α‐ω‐[NPR_2_]_*n*_ and DMPA as initiator in anhydrous THF and irradiated in UV‐light for 24 h at 4 °C. The direct radical polymerization was performed in anhydrous THF as well, however, initiated via AIBN and reacted for 24 h at 65 °C in a water bath. Both methods resulted in an insoluble crosslinked organogel, as can be seen in Figure 3, and served as a proof‐of‐principle for the availability of the end‐groups in the polymers for gel formation.

## 4 CONCLUSION

In conclusion, a versatile synthesis route to homo and hetero α,ω‐chain‐end‐functionalized poly(dichloro)phosphazenes is described. Upon macromolecular substitution, stable poly(organo)phosphazenes can be achieved as either a homo‐telechelic polymer with two styrene functionalities or a hetero telechelic polymer with one styrene and one protected benzoic acid moiety at their chain ends. The styrene functionalities at either one or both ends can be further converted via a thiol‐ene “click”‐reaction, as was shown by the formation of a macromolecular diol or an amine. The applicability of the synthesized homo chain‐end functionalized polymer for the development of organogels was demonstrated in preliminary experiments. This procedure thus represents a robust and versatile route to α‐ω‐[NPCl_2_]_*n*_, which can be readily functionalized along their main chain, and orthogonally at the chain ends, to a wide range of polymers. This method adds not only a new and robust route to block polyphosphazene copolymers but also as a versatile building block for the preparation of advanced soft materials.

## CONFLICT OF INTEREST

The authors declare no potential conflict of interest.

## Supporting information


**Data S1.** Supporting information.

## Data Availability

The data that supports the findings of this study are available in the supplementary material of this article.
