# Supplementary material for: Hetero and homo α,ω‐chain‐end functionalized polyphosphazenes
Source: J Polym Sci (2020). 2022 Apr 8;60(13):2000–7. doi: 10.1002/pol.20220066 (PMC9325445; doi:10.1002/pol.20220066)
Supplement: Supplementary file 1 — Data S1. Supporting information. [file POLA-60-2000-s001.docx]

Supporting Information

Hetero and homo α,ω-chain end functionalized polyphosphazenes

Paul Strasser, Oliver Plavcan, Edip Ajvazi, Helena Henke, Oliver Brüggemann, Ian Teasdale*

1. NMR-Spectra:

## **1.1 End-Capper 1:**

**Figure S1.** ^1^H-NMR spectrum in CDCl_3_ of the end-capper **1**.

##

**Figure S2.** ^31^P-NMR spectrum in CDCl_3_ of the end-capper **1**.


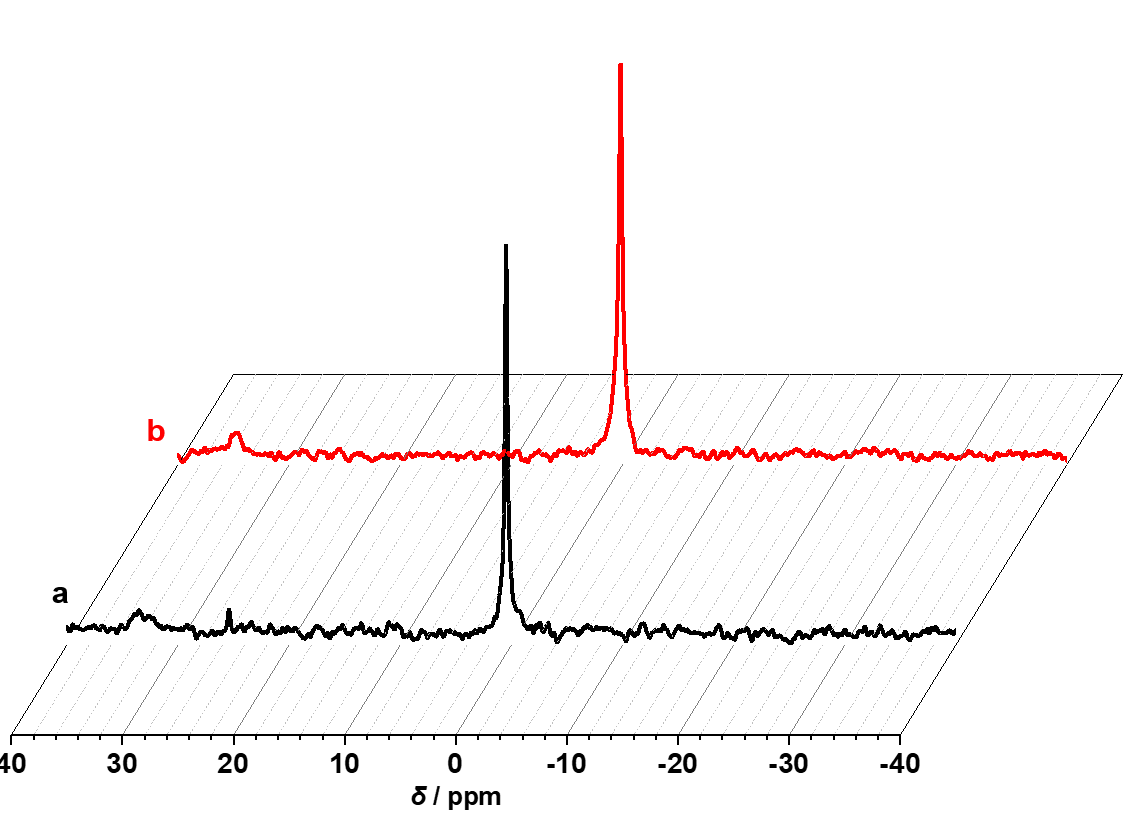


**Figure S3.** ^31^P-NMR spectra in CDCl_3_ of the end-capper (**1**) after (a) 1 day and (b) 4 months in diglyme.

## **1.2 Homo‑α,ω‑chain end functionalized poly(N‑(3‑aminopropyl)morpholine)phosphazene P3:**

**Figure S4.** ^1^H-NMR spectrum in CDCl_3_ of the homo α,ω-chain end functionalized poly(N‑(3‑aminopropyl)morpholine)phosphazene **P3**.

**Figure S5.** ^31^P-NMR spectrum in CDCl_3_ of the homo α,ω-chain end functionalized poly(N‑(3‑aminopropyl)morpholine)phosphazene **P3**.

## **1.3 Hetero‑α,ω‑chain end functionalized poly(N‑(3‑aminopropyl)morpholine)phosphazene P6:**

**Figure S6.** ^1^H-NMR spectrum in CDCl_3_ of the hetero α,ω-chain end functionalized poly(N‑(3‑aminopropyl)morpholine)phosphazene **P6**.

**Figure S7.** Zoomed ^1^H-NMR spectrum in CDCl_3_ of the hetero α,ω-chain end functionalized poly(N‑(3‑aminopropyl)morpholine)phosphazene **P6** showing the small signals of the styrene double bonds.

**Figure S8.** ^31^P-NMR spectrum in CDCl_3_ of the hetero α,ω-chain end functionalized poly(N‑(3‑aminopropyl)morpholine)phosphazene **P6**.

2. SEC-Data:

## **2.1 Hetero‑α,ω‑chain end functionalized poly(N‑(3‑aminopropyl)morpholine)phosphazene P6:**

**Figure S9.** Size exclusion chromatogram of the hetero α,ω‑chain end functionalized poly(N‑(3‑aminopropyl)morpholine)phosphazene **P6**.

3. Additional NMR-Data:

## **3.1 N-(trimethylsilyl)-trichlorophosphoranimine (Cl_3_PNSiMe_3_):**

**Figure S10.**^1^H-NMR spectrum in CDCl_3_ of the PPz-monomer Cl_3_PNSiMe_3_.

**Figure S11.** ^31^P-NMR spectrum in CDCl_3_ of the PPz-monomer Cl_3_PNSiMe_3_.

## **3.2 Homo‑α,ω‑chain end functionalized poly(N‑(3‑aminopropyl)morpholine)phosphazene P3:**

**Figure S12.** ^31^P-NMR spectrum in CDCl_3_ of 4-(diphenylphosphino)styrene.

**Figure S13.** ^31^P-NMR spectrum in CDCl_3_ of **2**.

**Figure S14.** ^31^P-NMR spectrum in CDCl_3_ of the α‑chain end functionalized poly(dichloro)phosphazene **P1**.

**Figure S15.** ^31^P-NMR spectrum in CDCl_3_ of the homo α,ω-chain end functionalized poly(dichloro)phosphazene **P2**.

## **3.3 Diol adapted homo‑α,ω‑chain end functionalized poly(N‑(3‑aminopropyl)morpholine)phosphazene P4:**

**Figure S16.** ^1^H-NMR spectrum in CDCl_3_ of the OH‑α,ω-chain end functionalized poly(N‑(3‑aminopropyl)morpholine)phosphazene diol **P4**.

##

**Figure S17.** ^31^P-NMR spectrum in CDCl_3_ of the OH‑α,ω-chain end functionalized poly(N‑(3‑aminopropyl)morpholine)phosphazene diol **P4**.

## **3.4 Amine capped hetero‑α,ω‑chain end functionalized poly(N‑(3‑aminopropyl)morpholine)phosphazene P7:**

**Figure S18.** ^1^H-NMR spectrum in CDCl_3_ of the amine capped hetero α,ω-chain end functionalized poly(N‑(3‑aminopropyl)morpholine)phosphazene **P7**.

**Figure S19.** ^31^P-NMR spectrum in CDCl_3_ of the amine adapted hetero α,ω-chain end functionalized poly(N‑(3‑aminopropyl)morpholine)phosphazene **P7**.
